# Ultrasound Versus Elastography in the Diagnosis of Hepatic Steatosis: Evaluation of Traditional Machine Learning Versus Deep Learning

**DOI:** 10.3390/s24237568

**Published:** 2024-11-27

**Authors:** Rodrigo Marques, Jaime Santos, Alexandra André, José Silva

**Affiliations:** 1 Faculdade de Ciências e Tecnologias, Department of Physics, University of Coimbra, Rua Larga, 3004-516 Coimbra, Portugal; digasman22@gmail.com; 2 Department of Electrical and Computers Engineering, CEMMPRE-ARISE, University of Coimbra, Polo II, Rua Sílvio Lima, 3030-970 Coimbra, Portugal; jaime@deec.uc.pt; 3 Polytechnic Institute of Coimbra, Coimbra Health School, 3046-854 Coimbra, Portugal; alexandra.andre@estesc.ipc.pt; 4 Military Academy Research Center (CINAMIL), Portuguese Military Academy, 1169-203 Lisbon, Portugal; 5 LIBPhys, LA-REAL, Faculdade de Ciências e Tecnologia, Universidade de Coimbra, 3004-516 Coimbra, Portugal

**Keywords:** machine learning, deep learning, image classification

## Abstract

The prevalence of fatty liver disease is on the rise, posing a significant global health concern. If left untreated, it can progress into more serious liver diseases. Therefore, accurately diagnosing the condition at an early stage is essential for more effective intervention and management. This study uses images acquired via ultrasound and elastography to classify liver steatosis using classical machine learning classifiers, including random forest and support vector machine, as well as deep learning architectures, such as ResNet50V2 and DenseNet-201. The neural network demonstrated the most optimal performance, achieving an F1 score of 99.5% on the ultrasound dataset, 99.2% on the elastography dataset, and 98.9% on the mixed dataset. The results from the deep learning approach are comparable to those of machine learning, despite objectively not achieving the highest results. This research offers valuable insights into the domain of medical image classification and advocates the integration of advanced machine learning and deep learning technologies in diagnosing steatosis.

## 1. Introduction

Steatosis, commonly referred to as fatty liver disease (FLD), is defined by the abnormal accumulation of fat within liver cells, specifically when it accounts for more than 5% of the liver’s total weight [1]. This condition is turning into a significant global health concern, since its prevalence is increasing, with estimates suggesting that 30% of the world’s population is being affected by it [2]. FLD can be divided into two categories according to its main cause: alcoholic fatty liver disease (AFLD) and non-alcoholic fatty liver disease (NAFLD). Both can originate from lifestyle factors and are strongly associated with type 2 diabetes, obesity, and metabolic syndrome [3]. When left untreated, NAFLD can progress into more serious liver diseases, such as non-alcoholic steatohepatitis (NASH), fibrosis, liver cancer, or even cirrhosis; this is why an accurate diagnosis is crucial for early intervention and effective management of the condition [4]. Medical imaging plays a pivotal role in the diagnosis and assessment of steatosis, offering non-invasive methods to evaluate liver fat content and liver stiffness. Since ultrasound is a widely accessible and cost-effective technique that can identify increased liver echogenicity, an indicative sign of steatosis, it is the main imaging modality used to detect FLD. However, elastography has emerged in this field, offering advanced assessment by measuring liver stiffness, which correlates with fibrosis and inflammation levels [5].

Machine learning (ML) and deep learning (DL) techniques have significant potential to improve the diagnostic capabilities of ultrasound and elastography imaging. By leveraging large datasets and sophisticated algorithms, ML and DL can improve image analysis, feature extraction, and pattern recognition, leading to more accurate classification of steatosis. These techniques can overcome the limitations of traditional imaging methods by providing automated, objective, and reproducible assessments, potentially transforming the clinical management of liver diseases [6].

This study aims to harness the power of artificial intelligence in the diagnosis of liver steatosis, and contributes to the following:

- Developing ML and DL models for the classification of steatosis using ultrasound and elastography imaging data.
- Comparing the performance of the ML algorithms and DL architectures in a medical image classification context.
- Investigating the potential of elastography as well as its combination with ultrasound imaging data in diagnosis auxiliary tools, such as AI algorithms.

## 2. Related Work

Various studies have proven the reliability of ultrasound in grading and identifying steatosis; it is the non-invasive gold standard when it comes to the diagnosis of FLD [7,8,9]. However, elastography has shown the potential to help in diagnosing liver diseases and the different types of this imaging technique are still being evaluated in terms of their trustworthiness in assessing and classifying liver steatosis and fibrosis [10,11,12,13]. Furthermore, in the past years, with the growth of artificial intelligence, some researchers have applied machine learning and deep learning techniques to aid in the diagnostic process of NAFLD and fibrosis. In this section, various studies that use DL and ML approaches to stage and help diagnose liver steatosis and fibrosis will be covered, using mostly ultrasound data but also elastography data.

### Machine Learning and Deep Learning Approaches

Tsung-Hsien Chou et al. [14] developed two models based on a ResNet50V2 architecture to assess the severity of fatty liver, with the first model classifying fatty liver into three categories (normal, mild, and moderate-to-severe), and the second model providing a more detailed classification into four categories (normal, mild, moderate, and severe). Both were trained using a dataset comprising 21,855 B-mode abdominal ultrasound images from 2070 patients. The three-class model outperformed the four-class model in the moderate-to-severe field, with the first one achieving an AUC of 99.6% and the second achieving AUC values of 97.1% and 98.1% for moderate and severe fatty liver, respectively. The ResNet50V2 achieved an overall F1 score of 84.0%. These results suggest that machine learning models may benefit from a three-class prediction system. However, the authors point out that, since they made predictions based on single, still images instead of serial images (whole sequence of images in a time frame) from a patient, meaning that only an isolated image was used for categorization, their model could have been too subject to erroneous classification due to motion artifacts and image variability.

A study conducted by Codruta Constantinescu et al. [15] employed the pre-trained Inception V3 model’s fine-tuning, which might identify non-alcoholic fatty liver disease more accurately than through feature extraction. The top layers were changed by adding completely connected layers with arbitrary parameters in place of the lower layers. The dataset used to train the model had 629 grayscale liver B-mode images, where 496 were used for training and the remaining 133 for testing. After scaling and employing picture augmentation, the model was trained with an image batch size of 16. To prevent overfitting, dropout, activity regularization, and kernel regularization were applied. The accuracy achieved by the Inception V3 was 93.23%, as well as it had a sensitivity of 88.9% and a precision of 96.6%.

In addition, another study by Byra et al. [16] used 550 B-mode scans and the biopsy results of 55 patients to constitute the dataset used to train an Inception-ResNet-v2 DCNN, pre-trained using the ImageNet dataset. Minimal pre-processing was applied, resizing the images to the network resolution using bi-cubic interpolation, and removing non-relevant data. The ResNet network uses texture features as input along with the ultrasound picture data to diagnose liver steatosis. Before the images were fed into the ResNet model, they were scaled to 512 × 512 pixels. Following this, the images were classified using the SVM algorithm. This methodology achieved a 93.8% accuracy and a 99.7% AUC with the Inception-ResNet-v2.

Bowen Li et al. [17] trained a multi-class deep ResNet-18 DL algorithm to diagnose steatosis stages (healthy, mild, moderate, or severe) using multiple perspective clinical ultrasound images from 3310 patients that underwent elastography, amounting to 228,075 pictures. In order to deal with the large amount of data, the deep learning algorithm was designed to be scalable. Additionally, the reliability of the model across different viewpoints was evaluated using Bland–Altman analysis, ensuring consistency from various perspectives. To measure diagnostic performance, the area under the receiver operating characteristic curve (AUC-ROC) metric was used; it showed 85% for mild steatosis, 91% for moderate steatosis, and 93% for severe steatosis. The algorithm demonstrated repeatable measurements, with three images per viewpoint, and high agreement across different ultrasound scanners, indicating that this deep learning algorithm is reliable in real-world clinical scenarios for steatosis diagnosis.

Some studies have been made using elastography as their main imaging method. George C Kagadis et al. [18] used a multi-step methodology to fine-tune shear wave elastography image sequences and evaluated the performance of various deep learning networks on the classification of chronic liver disease stages. The authors examined 200 patients—112 with liver biopsy-validated chronic liver disease and 88 healthy individuals. For each patient, four shear wave elastography images from the same liver area were extracted and then pre-processed. Using clustering techniques and wavelet transforms, significant differences in stiffness, over time, were tracked and used to generate a mask that defined areas according to their temporal stability. This allows for the isolation and detection of areas with varying degrees of stiffness over time and was implemented to improve the accuracy of diagnosis. Five networks were trained using the pre-processed image sequences, and different input combinations were tested, including single-frame and multi-frame inputs as well as with and without temporal stability masks. All networks achieved AUCs ranging from 0.979 to 0.990, which were higher than the radiologists who went through the evaluation of all the images. The ResNet50 and DenseNet-201 architectures showed the best results with accuracies of 98.9% and 98.7%, respectively. Half of the networks demonstrated an improvement in classification when using temporal stability masks, particularly those that achieved the best results. The authors also noted that using data augmentation generally led to lower performance. In conclusion, the usage of shear wave elastography to differentiate stages of chronic liver disease shows promise, but the employment of a larger dataset would be advised to confirm these findings.

## 3. Materials and Methods

**Figure 1:**
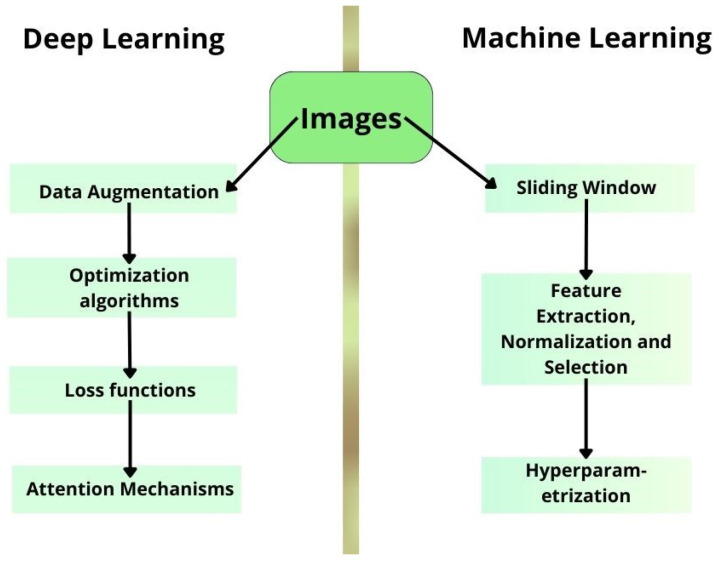
Diagram with the main stages of this work.

The stages of this work are summarized in the diagram in Figure 1. The methodology adopted in this research combines distinct approaches for both deep learning and traditional machine learning. The machine learning approach begins with a sliding window technique, which is employed to augment the number of images. Subsequently, the data undergo a series of operations, including feature extraction, normalization, and selection, which are collectively designed to prepare and refine the dataset, ensuring that only the most relevant features are used for modeling purposes. The final stage of the process entails the fine-tuning of the algorithmic settings through hyper-parameterization, thereby optimizing the model’s performance. In the context of deep learning, the initial step involves data augmentation, which serves to enhance the training dataset. This is followed by the application of optimization algorithms, which are used to adjust the model parameters to minimize errors in classification. The performance of the model is then evaluated using specific loss functions, which are employed to quantify errors and guide the learning process. Finally, attention mechanisms are employed to focus on the most relevant parts of the input data, thereby improving the model’s accuracy.

### 3.1. Datasets

The data used in this study were obtained from two sources, one being the public dataset of B-mode fatty liver ultrasound images [16], and the other one being a private dataset of images acquired at the Coimbra Health School.

The dataset comprises 550 B-mode grayscale images of the liver—380 of which were obtained from patients with steatosis and 170 from healthy individuals. It is part of a study by Byra et al. [16] that included 55 severely obese patients with a mean age of 40.1 ± 9.1 years. Of these patients, 44 were females and 11 were males. The ultrasound images were obtained using a GE Vivid E9 system, equipped with a phased array probe operating at 2.5 MHz, resulting in images with a resolution of 434 × 636 pixels. Each patient underwent an acquisition of 10 images in sequence.

**Figure 2:**
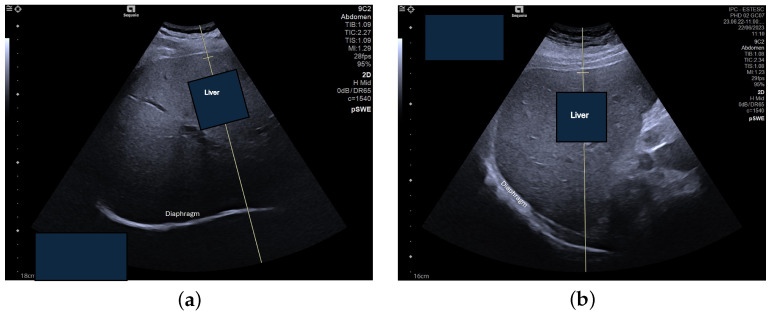
Mode B intercostal scan of the right lobe of the liver, acquired with the Acuson Sequoia ultrasound system, showing the liver: (**a**) without steatosis, (**b**) with steatosis.

**Figure 3:**
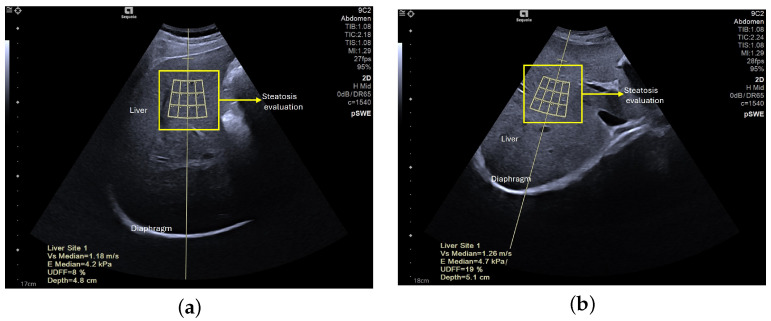
Intercostal scan of the right lobe of the liver, acquired with the Acuson Sequoia ultrasound system equipped with Auto pSWE and ultrasound-derived fat fraction (UDFF) technique. The inner rectangle is the fixed measurement box: (**a**) UDFF = 8% (no steatosis), (**b**) UDFF = 19% (steatosis).

The second set of images acquired for this study is composed of 96 elastography images—61 of NAFLD patients and the remaining 35 of healthy subjects. These were obtained at the School of Health Technology from Coimbra, extracting data from 50 participants, ranging from 18 to 50 years old. A consistent procedure was used to acquire the images for the dataset. Each subject was studied by a single operator who performed fist the US assessment of the liver steatosis in B-mode. A portable ultrasound (Acuson P500) with a convex transducer (5.2 MHz), was operated by one experienced researcher (>20 years). The collection was conducted during rest. Shear-wave elastography (SWE) data collection was conducted using the abdomen shear module of an Acuson Sequoia Ultrasound System 2018; this device was coupled with a convex transducer (9C2 Mhz). Figure 2 and Figure 3 are examples of acquired images. In order to maintain consistency across all acquisitions, a quality factor of over 65 was consistently achieved, enhancing the accuracy of classification and analysis, as well as image quality. The settings were maintained at a frame rate of 27 fps (frames per second) and a vertical scale (V scale) of 95%.

The two sets of images were used to train and test the models, dividing them into three different datasets: Dataset A, with the ultrasound images; Dataset B, with the elastography images; and Dataset C, which uses images from both datasets. As the classes for classification had a 70/30 ratio, the number of images in the ’Steatosis’ class was reduced, randomly undersampling to a number equal to the number of images in the ’Normal’ class.

### 3.2. Data Preprocessing and Augmentation

Firstly, due to the limited number of images for training and testing all the DL and ML models, there was a necessity to increase this number. To address this issue, two techniques were utilized—one for the classic ML approach and the other for the DL approach.

In the ML approach, a sliding window technique was used to extract various secondary regions of interest from the original images. For the DL approach, a data augmentation pipeline consisting of random Gaussian noise and geometric transformations such as rotation, scaling, and mirroring operations was applied to the data.

The next step involved feature extraction, normalization, and selection. The images resulting from the sliding window technique were first opened and stored as an RGB matrix and immediately converted to grayscale. Using these grayscale images, statistical, textural, morphological, and shape-related features were extracted. To ensure all the features are on the same scale, a normalization algorithm (StandardScaler) was used, followed by the selection of the k-best features to feed the models, using the SelectKBest function. Since the DL approach utilized convolutional neural networks, the feature extraction step was performed directly by the model.

### 3.3. Machine Learning Classifiers

In this study, we employ several machine learning models to efficiently analyze and predict outcomes based on our dataset. This section is dedicated to briefly describing each of the six classifiers utilized.

#### 3.3.1. Decision Tree

Decision trees are supervised learning algorithms that create a tree-like model of decisions and their possible outcomes. They work by recursively partitioning the data based on the feature that provides the most information gain (IG), creating a hierarchical structure of decisions [19], where the decision at each node is based on a criterion such as IG or Gini impurity. The Gini impurity for a dataset *D* is defined in Equation (1), as follows:

(1) $$\begin{array}{c} Gini\left(D\right)=1-\sum_{i=1}^{n}p_{i}^{2} \end{array}$$

where $p_{i}$ is the probability of class *i* in dataset *D*. These classifiers are intuitive, easy to interpret, relatively quick to train, and can handle large datasets.

#### 3.3.2. Gradient Boosting Classifier

The gradient boosting algorithm, which is an ensemble algorithm, combines several “weak” models (decision trees by default) and iteratively trains new ones correcting the errors of the previous models and gradually improving overall performance [20]. Equation (2) shows that, in this classifier, a model $F_{m}\left(x\right)$, at stage *m* is updated as follows:

(2) $$\begin{array}{c} F_{m}\left(x\right)=F_{m-1}\left(x\right)+\gamma_{m}h_{m}\left(x\right) \end{array}$$

where $h_{m}\left(x\right)$ is the *m*-th tree fitted to the negative gradient of the loss function $L(y,F_{m-1}\left(x\right))$, and $\gamma_{m}$ is the step size. Gradient boosting is known for its ability to handle complex, non-linear relationships and its robustness to outliers.

#### 3.3.3. K-Nearest Neighbors

K-nearest neighbors (KNN) is a simple algorithm that classifies a data point based on the majority class of its k-nearest neighbors in the feature space. It is a lazy learning algorithm, meaning that it does not require a training phase and instead performs the classification at the time of prediction, being relatively insensitive to outliers and dealing effectively with non-linear decision boundaries [21]. The predicted class $\hat{y}$ for a point *x* is computed as in Equation (3):

(3) $$\begin{array}{c} \hat{y}=\arg\max_{c\in C}\sum_{i\in N_{k}\left(x\right)}I\left(y_{i}=c\right) \end{array}$$

where $N_{k}\left(x\right)$ is the set of *k* nearest neighbors to *x*, $y_{i}$ is the class of neighbor *i*, and *I* is the indicator function.

#### 3.3.4. Neural Network

Neural networks are a class of machine learning models inspired by the structure and function of the human brain. Consisting of interconnected nodes (neurons) that learn patterns in the data through a training process, neural networks are highly flexible and can model complex, non-linear relationships, making them suitable for a wide range of classification tasks [22]. In addition, they are also very effective at handling high-dimensional data. Equation (4) represents the general equation for a single neuron in a fully connected layer as follows:

(4) $$\begin{array}{c} a_{j}^{\left(l\right)}=f\left(\sum_{i}w_{ij}^{\left(l\right)}a_{i}^{\left(l-1\right)}+b_{j}^{\left(l\right)}\right) \end{array}$$

where $a_{j}^{\left(l\right)}$ is the activation of neuron *j* in layer *l*, $w_{ij}^{\left(l\right)}$ is the weight from neuron *i* in layer l−1 to neuron *j* in layer *l*, $b_{j}^{\left(l\right)}$ is the offset of neuron *j* in layer *l*, and *f* is the activation function (e.g., sigmoid, ReLU).

#### 3.3.5. Random Forest

Random forest is also an ensemble learning algorithm that combines multiple decision trees to create a more robust and accurate classifier. It works by training each decision tree on a random subset of the features and joining the predictions of the individual trees [23]. For classification, the random forest prediction $\hat{y}$ for an input *x* is as seen in Equation (5):

(5) $$\begin{array}{c} \hat{y}=\mathrm{mode}\left\{T_{i}\left(x\right)\;|\;i=1,2,\ldots ,N\right\} \end{array}$$

where *N* is the number of trees in the forest, and $T_{i}\left(x\right)$ is the prediction of the *i*-th tree. This classifier is known for its ability to handle high-dimensional data, its robustness to overfitting, and its interpretability.

#### 3.3.6. Support Vector Machine

Support vector machine (SVM) is a powerful algorithm for both binary and multi-class classification problems. It can use different kernels to find the optimal hyperplane that separates the classes with the maximum margin [24]. The radial basis function (RBF) kernel used in this work is a flexible version of this algorithm that can handle non-linear decision boundaries, allowing the model to learn complex, non-linear relationships in the data and making it suitable for a wide range of classification problems.

The decision function for a non-linear SVM using a kernel *K* is represented by Equation (6):

(6) $$\begin{array}{c} f\left(x\right)=\sum_{i=1}^{N}\alpha_{i}y_{i}K\left(x_{i},x\right)+b \end{array}$$

where $\alpha_{i}$ are the Lagrange multipliers, $y_{i}$ are the training labels, and $K(x_{i},x)$ is the kernel function.

### 3.4. Deep Learning Architectures

Our methodology also incorporates advanced deep learning architectures to capture intricate patterns and relationships within the dataset, also enhancing predictive performance. All five architectures used in this work were pre-trained using the ImageNet dataset [25], allowing knowledge to be transferred from the source task to the target task, just by adding a new classifier layer to satisfy the new task [26].

#### 3.4.1. DenseNet-201

DenseNet-201 is part of the DenseNet family of models, which are characterized by their unique form of connectivity between layers, where each layer in the network is directly connected to every other layer in a feed-forward fashion. The network begins with a standard convolutional layer, followed by a max-pooling layer to reduce the spatial dimensions of the input. Being the core of the DenseNet architecture, a series of four dense blocks with 32 convolutional layers each, are interspersed with transition layers where the number of feature maps and spatial dimensions of the input is reduced by using both an average pooling and a 1 × 1 convolutional layer. At the end of the network, there is a global average pooling layer used to aggregate the feature maps, followed by a fully connected layer for classification [27].

Its efficient feature reuse from earlier layers can lead to more effective feature extraction and a reduction in the number of parameters required to train a model, compared to traditional CNN architectures. The dense connections can improve the flow of gradients, helping to mitigate the vanishing gradient problem that occurs in deep neural networks. Also, because of its unique connections paired with the use of global average pooling, this network encourages the model to learn more transferable and generic features, helping in the reduction of overfitting.

#### 3.4.2. Inception-ResNet-v2

Inception-ResNet-v2 is a deep neural network architecture designed for handling image classification tasks. Combining the strengths of both Inception and ResNet architectures, it consists of several key components [28]. Inception blocks are the fundamental elements of the inception architectures and are composed of multiple parallel branches, each with a different filter size. This allows the network to capture features at different scales and resolutions. The residual connections, a ResNet architecture feature, allow the network to learn residual functions, which are the differences between the input and output of each block, helping to ease the training process and improve the overall performance of the network. Composed of several convolutional and pooling layers, the stem block represents the initial stage of the network, responsible for extracting low-level features from the input image. Auxiliary classifiers are used to classify the output of each inception block, which helps to improve the overall performance of the network [28].

The Inception-ResNet-v2 architecture demonstrates an enhanced performance and a higher degree of flexibility when compared to other approaches, its combination of the strengths of the Inception and ResNet architectures enables it to achieve state-of-the-art performance on a wide range of image classification benchmarks. Its typical residual connections and auxiliary classifiers facilitate the training process, enhancing its efficiency and effectiveness.

#### 3.4.3. ResNet50V2

A residual network (ResNet) is a deep neural network architecture created to alleviate the problem of vanishing gradients in deep neural networks [29]. The whole architecture is based on the idea of residual learning, where the output of each layer is added to the input of the next layer, allowing the network to learn the residual function between the input and output of each layer, rather than learning the entire function. This allows the network to learn more complex functions and helps to mitigate the vanishing gradient. Consisting of several convolutional layers, a batch normalization layer, and a ReLU activation function, the residual block is the basic building block of the ResNet architecture. These blocks are connected via residual connection, which is the key component that allows the network to learn residual functions. It works by connecting the output of each residual block to the input of the next block. Downsampling layers, such as max-pooling, are used to reduce the spatial dimensions of the input data, which helps to reduce the number of parameters and the computational cost of the network [29].

The residual connections help to alleviate the vanishing gradient problem, making it easier to train deep neural networks, and its ability to learn residual functions allows it to learn more complex functions, resulting in better performance on various tasks.

#### 3.4.4. VGG16 and VGG19

The VGG (visual geometry group) architecture is a deep convolutional neural network model developed by researchers at the University of Oxford. Known for its simplicity and consistent performance across a wide range of computer vision tasks, this architecture is characterized by the use of small convolutional filters and max-pooling layers sequentially stacked to form the network.

VGG architecture uses a series of 3 × 3 convolutional layers stacked on top of each other, being able to capture more complex features by increasing the depth of the network while keeping the number of parameters relatively small compared to larger filter sizes. After every few convolutional layers, 2 × 2 max-pooling layers are used. These layers reduce the spatial dimensions of the feature maps, effectively downsampling the input and allowing the network to learn more abstract representations. The final stages of the VGG architecture consist of three fully connected layers that take the flattened feature maps from the convolutional and pooling layers and produce the final classification output [30].

The VGG16 and VGG19 models are two of the most popular variants of the VGG architecture. The main differences between these are their depth and number of parameters. VGG16 has 16 convolutional layers, while VGG19 has 19 convolutional layers, the additional layers in VGG19 allow the network to learn more complex features but also increase the complexity of the model and the number of parameters. VGG16 has approximately 138 million parameters, while VGG19 has approximately 144 million parameters [30]. Both have demonstrated strong performance on various computer vision tasks, with VGG19 generally achieving slightly higher accuracy than VGG16 due to its increased depth and capacity.

### 3.5. Loss Functions

Loss functions provide a measure of the effectiveness of the model’s operation by quantifying the difference between the predicted results and the true labels. These work by optimizing the model training process, guiding the algorithm to reduce errors and improve model predictions [31]. Deep learning models include loss functions as a fundamental component, particularly for classification-based tasks.

A common loss function for classification problems is the cross-entropy loss, which aims to look for discrepancies between the model predictions and the actual classes in the dataset. The deviation between the probability predicted by the model and the ideal probabilities associated with the correct classes is calculated as the cross-entropy loss. The model produces more accurate predictions, which are closer to reality, when the difference between the correct probabilities and the predicted probabilities is smaller, resulting in a lower loss value [32].

A special loss function, called focal loss, has been developed to deal with the imbalance and the problem of hard-to-classify samples in classification tasks. It adds a modulator element that reduces the contribution of the loss from correctly categorized instances, giving more attention to problematic samples [33]. Focal loss encourages the model to focus on difficult examples, reducing the influence of simple negative data, and thereby improving overall performance.

Therefore, in this work, the performance of the models when cross-entropy loss and focal loss functions were applied was compared.

### 3.6. Optimization Algorithms

Optimization algorithms are essential for training deep learning models to reduce the loss function and improve performance. Iteratively updating the parameters of a model based on the gradients of the loss function with respect to these parameters is the goal of an optimization procedure. The goal of this algorithm is to identify the optimal set of parameters that minimize the loss and enhance the model’s generalization to new data.

Three optimization algorithms were implemented for comparison of their effects on model performance: Adam, SGD, and RMSProp.

By calculating exponential moving averages of the gradient (first moment) and the squared gradient (second moment), the Adam optimizer maintains an adaptive learning rate for each parameter. In order to provide reliable estimates, bias correction is also included. The system modifies the learning rate depending on the estimated first and second moments, which allows it to adapt to different learning rate needs for various parameters and converge more quickly. Adam is known for its reliability, efficiency, and strong performance in a variety of deep learning tasks, hence why it was chosen initially [34].

The SGD algorithm modifies the model parameters based on the gradients of the loss function calculated on small batches of training data. Using the chosen batch, it determines the gradients of the loss function concerning the parameters and updates them by deducting the learning rate multiplied by the gradients, repeating this process iteratively until a convergence condition is met, which can take several epochs. Although it is straightforward and simple to use, it can be sensitive to the learning rate, and can converge slowly or become stuck in non-ideal solutions [35].

The RMSProp optimizer is an algorithm that adapts the learning rate for each parameter based on the magnitude of the gradient. It does this by maintaining a moving average of the squared gradients and dividing the learning rate by this average, allowing the algorithm to dynamically adjust the learning rate, helping it to converge faster and avoiding being stuck in local minima. RMSProp is particularly effective in deep neural networks where the gradients can be highly variable and are often used in combination with other optimization techniques such as momentum and Nesterov acceleration [36].

### 3.7. Attention Mechanisms

Deep learning models can selectively focus on the most relevant parts of the input, thanks to the development of attention mechanisms. Models can use attention processes to give different levels of emphasis to certain areas or aspects of an input. Attention mechanisms improve the model’s ability to recognize important patterns and make accurate predictions by selectively attending to instructive regions [37]. This is particularly useful in the classification of medical images, where it is crucial to locate and identify specific features of pathology. In this project, the multi-head attention mechanism was used to understand its relevance.

Multi-head attention is a mechanism used in neural networks to process and combine information from different sources. It works by dividing the input into multiple parallel attention mechanisms, each of which focuses on a different aspect of the input. These parallel mechanisms then combine their outputs through a weighted sum, where the weights are learned during training. This allows the network to capture complex relationships between different parts of the input. The benefits of multi-head attention include an improved ability to capture long-range dependencies and to handle high-dimensional inputs. However, it can also lead to increased computational complexity and may not always be effective for tasks where the relationships between different parts of the input are simple or straightforward [38].

### 3.8. Performance Metrics

Three commonly used metrics (precision, recall, and F1 score) were chosen for this work. These metrics are based on four main results that can occur when comparing the model’s predictions with the actual values. These results are commonly known as true positives (*TPs*), i.e., samples correctly predicted as positive, false positives (*FPs*), i.e., samples predicted to be positive but are actually negative), true negatives (*TNs*), i.e., samples correctly predicted as negative), and false positives (*FPs*), i.e., samples predicted as negative but are actually positive).

(7) $$\begin{array}{c} \mathrm{Precision}=\frac{TP}{TP+FP} \end{array}$$

In classification problems, precision is an evaluation metric that measures the proportion of examples that were correctly classified as positive, in relation to the total number of examples classified as positive by the model [31]. It can be determined by using Equation (7).

(8) $$\begin{array}{c} \mathrm{Recall}=\frac{TP}{TP+FN} \end{array}$$

Represented by Equation (8), recall, also known as the sensitivity rate or true positive rate (TPR), is an evaluation metric that calculates the percentage of positive examples correctly identified compared to the total number of positive examples [31].

(9) $$\begin{array}{c} F1\;\mathrm{score}=\frac{TP}{TP+\frac{1}{2}\left(FP+FN\right)} \end{array}$$

The F1 score, described by Equation (9), is an evaluation metric that combines recall and precision into a single value, to provide a broader evaluation of the performance of the classification model [31].

## 4. Results and Discussion

The experimental work was carried out in Google Colab, using Python, to develop and test the deep learning and machine learning models.

### 4.1. Classical Machine Learning Approach

Six different classifiers (decision tree, gradient boosting, KNN, neural network, random forest, and SVM) were trained, testing various numbers of features as well as sliding window and step sizes, and comparing the performances of the trained models.

A sliding window algorithm with a window size of 35 × 35 pixels and a step size of 13 pixels was applied to Datasets A, B, and C. The features were normalized using the standard scaling algorithm, and 50 features were selected using the Select K-Best algorithm. To split the data, a 10-fold algorithm was applied, it provides indices that randomly divide the data into k subsets (10 in this case), each subset being tested once as test data and used as training data in the remaining iterations. The six models were compared while using the parameters resulting from the hyperparameter optimization process with the Bayesian optimization function. The parameters used are shown in Table 1.

#### 4.1.1. Number of Features

After obtaining the best possible parameters through hyperparameter optimization, the performance of each classifier was compared for several features between 5 and 100, in increments of 5, using the SelectKBest feature selection algorithm.

**Figure 4:**
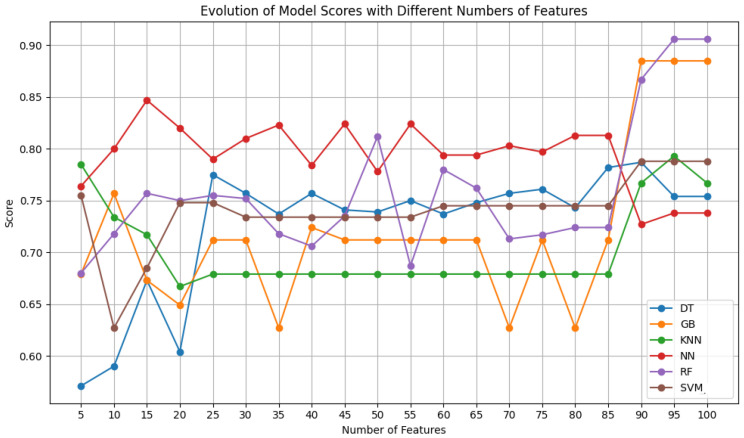
F1 score of the selected classifiers, for a variety of features, using Dataset A.

Figure 4 shows that for the ultrasound dataset, there is an improvement in results from 90 features onward for the decision tree, gradient boosting, KNN, random forest, and SVM models. The only exception is the neural network, which achieves the best results with 15 features. Initially, its performance oscillates significantly but becomes more stable up to 90 features, beyond which the performance visibly worsens. The neural network is the classifier with the best results for the smallest number of features, and its performance only drops when the other classifiers improve (90 to 100 features). This trend confirms that neural networks do well with fewer features, as they are less prone to overfitting. The noticeable drop in their performance for 90 features or more highlights the increase in network complexity and greater adaptation to the test data, which hampers their ability to generalize well. The other classifiers are more robust to this phenomenon, especially random forest and gradient boosting, which operate in a manner opposite to that of neural networks. Both utilize decision trees, which are highly capable of managing high-dimensional feature spaces and capturing increasingly complex non-linear relationships as the number of features increases.

**Figure 5:**
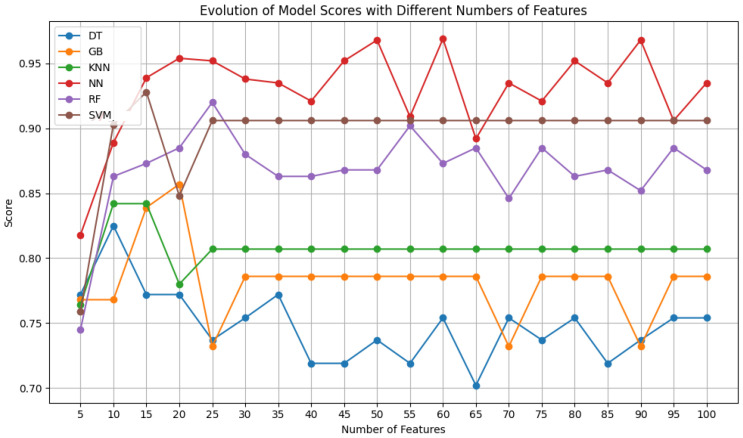
F1 scores of the selected classifiers for a variety of features, using Dataset B.

Contrary to the results obtained with Dataset A, the models trained with Dataset B, as can be seen in Figure 5, show an improvement from the beginning of the feature variation and achieve the best results with fewer features, generally between 10 and 30 features. The inversion of the pattern seen with Dataset A could be due to the fact that the elastography images need fewer features to be classified correctly, so the models do not improve after a certain point. Once again, the neural network is the exception, although it is the best model for almost all the numbers of features used, it reaches its peak at 60 features. The RF and SVM classifiers with an RBF kernel, together with NN, continue to obtain the best results and begin to appear as the best classifiers in this specific context.

**Figure 6:**
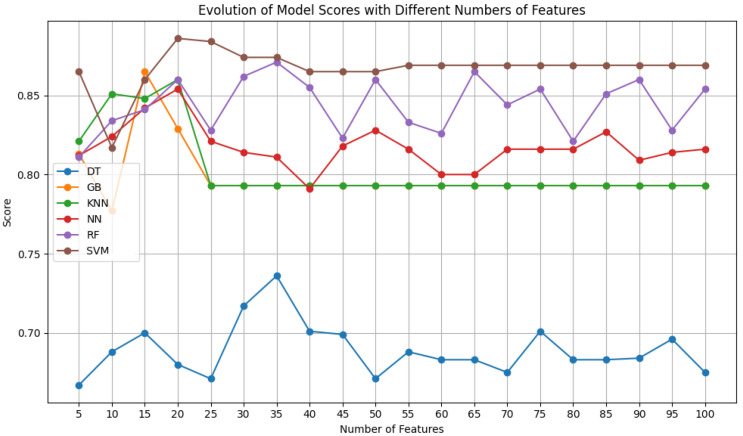
F1 scores of the selected classifiers for a variety of features, using Dataset C.

For the set that combines images from both imaging methods, it should be noted that all the classifiers show their best results between 20 and 35 features, with no major performance fluctuations for a larger number of features. The combination of two types of images provides a more comprehensive and balanced representation, making the models more stable and reaching their peak with a more moderate number of features. As seen in the previous figures, Figure 6 shows the superior performance of the NN, RF, and SVM models; moreover, gradient boosting emerges as the best classifier for 15 features, achieving a higher F1 score than the NN in that instance.

Since Dataset A required a larger number of features to achieve better performance, while the other two datasets (B and C) achieved it with fewer features and remained stable with a larger number, a moderate number of features, 50, was chosen for training across all datasets. This number is not too high, helping to avoid overfitting. The choice of standardizing the number of features chosen for the three datasets was made so the comparisons made onward were simplified, as establishing a uniform baseline makes it easier to benchmark and assess the relative performance of the classifiers.

#### 4.1.2. Window and Step Size in Sliding Window

**Figure 7:**
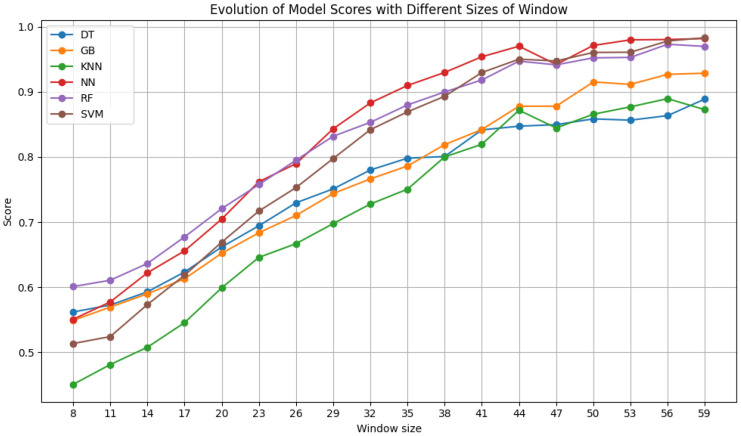
F1 scores of the selected classifiers for varying window sizes with a step size of 6, using Dataset A.

**Figure 8:**
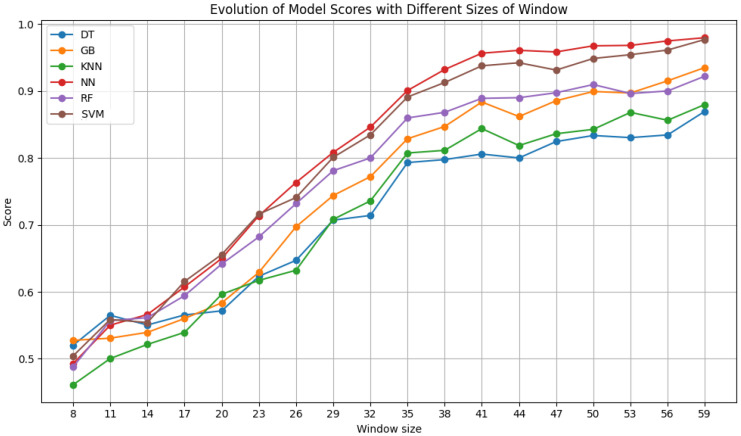
F1 scores of the selected classifiers for varying window sizes with a step size of 6, using Dataset B.

**Figure 9:**
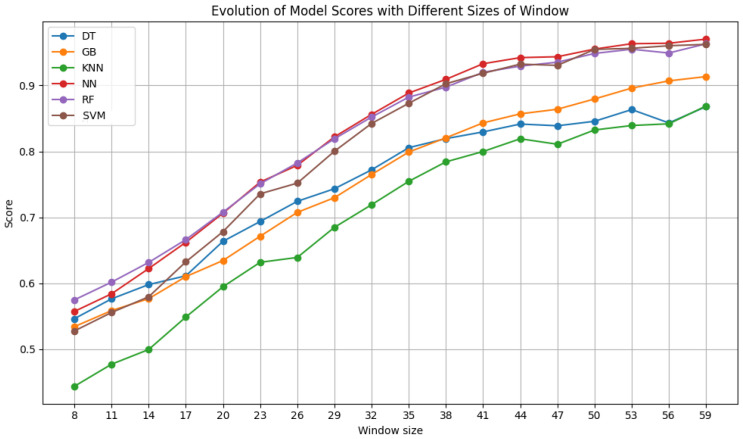
F1 scores of the selected classifiers for varying window sizes with a step size of 6, using Dataset C.

**Figure 10:**
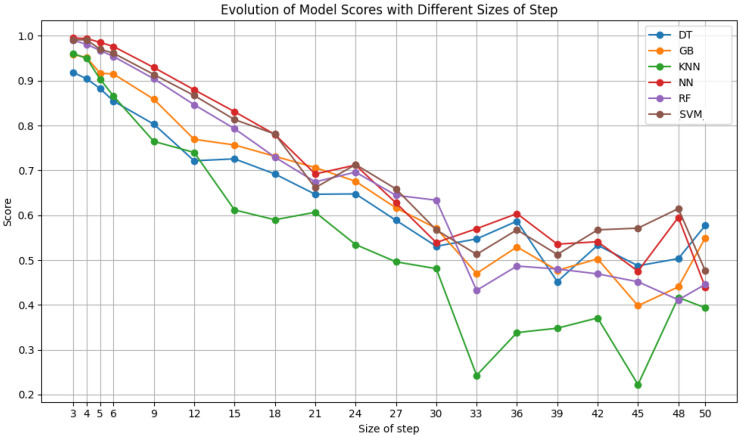
F1 scores of the selected classifiers for a window size of 50 pixels with variable step sizes, using Dataset A.

After testing the ideal number of features, additional tests were made to understand the influence of window size and step size of the sliding window algorithm on the results. The window size ranged from 8 × 8 pixels to 59 × 59 pixels in increments of 3 pixels. The step size was varied between 3 and 50, in increments of 3, never exceeding the window size. The results of each classifier for varying the window size with a fixed step size of 6 are shown in Figure 7, Figure 8 and Figure 9. Moreover, the results for varying the step size with a fixed window size of 50 × 50 pixels are shown in Figure 10.

Figure 7 shows the results obtained by the models on Dataset A. There is a clear improvement in the performance of all the classifiers as the window size increases, reaching their peaks starting at 50 pixels. From 44 pixels onward, the performance improvement becomes less marked, so the models do not improve as much thereafter. The results of the NN, RF, and SVM with RBF classifiers all exceed a 90% F1 score for sizes of 41 pixels or more. Gradient Boosting consistently surpasses this F1 score level at sizes of 47 pixels or more.

Figure 8, for Dataset B, reveals that larger window sizes yield superior outcomes. The improvement is no longer as significant after 41 pixels for all the classifiers, meaning that from this size onward, the results differ less and less from each other. This occurs likely due to the introduction of redundant information. Starting at a size of 38, both NN and SVM always surpass the 90% F1 score mark, with RF and gradient boosting joining them after sizes of 44 and 50, respectively.

As with the previous dataset, the performance of all the classifiers improves with an increase in step size for Dataset C (Figure 9). For the most effective classifiers, the improvement trend reaches its maximum at the largest size that was tested, 59 × 59 pixels. Once again, the RF, NN, and SVM classifiers with RBF kernel demonstrate consistent superiority, consistently surpassing the 90% F1 score threshold from sizes of 38 × 38. Gradient boosting only attains a similar level of performance with windows of 56 × 56 and 59 × 59 pixels. This general trend of improved performance with larger window sizes is understandable, as a larger input contains more characteristics that can aid in identifying and classifying data, allowing for the incorporation of more varied information. However, the decline in results beyond a certain point is also explainable, as including more information might also introduce irrelevant or redundant data, which the results reflect. For this reason, to avoid excess information that could lead to overfitting and increased model complexity, window sizes larger than 59 × 59 were not tested, even though slight improvements in results could possibly happen.

Figure 10, which depicts the outcome of varying the step size using Dataset A, illustrates that the trend is in opposition to that observed with the window size. Therefore, all the classifiers exhibited optimal performance at 3 and 4 pixels; however, as we increase this parameter, the results become worse, reaching 27 pixels, where the performance becomes very unstable and has large oscillations. An increase in step size results in a reduction in the quantity of data, which is reflected in the absence of data up to 50 pixels, where the results are no longer reliable or satisfactory. In particular, the NN, RF, and SVM RBF classifiers exhibited a remarkable performance, approaching an F1 score of 100%.

Evaluating the results of the step variation using Dataset B, there is still a peak in performance for steps between 3 and 5 pixels, with a subsequent decrease and an oscillation in the results for a step greater than 24. In this instance, only the NN and SVM classifiers approach 100% of the metric, with gradient boosting also approaching an F1 score of 95%, similar to RF.

Dataset C, which combines ultrasound and elastography images, exhibits a similar trend to the aforementioned in this variation, with enhanced outcomes for smaller strides, a decline in performance, and an increase in model instability and lack of robustness as the stride size increases. Similarly, with Dataset C, every classifier exceeds an F1 score of 90% when using a step size of 3. It can be said that, in relation to the step size test, as the amount of data present in each set varies, a smaller step size results in a greater quantity of data, thereby enhancing the robustness and accuracy of the models. In contrast, a larger step size leads to a reduction in data, which in turn affects the stability and precision of the model’s performance. Furthermore, a smaller step size guarantees that the models are trained with more consistent sets of features, thereby enhancing their robustness to alterations in the data and ensuring more reliable and stable learning of their patterns.

#### 4.1.3. Final Evaluation

The best performances of each classifier, obtained with the combination that optimizes each of them, for the three datasets, are shown in Table 2.

The neural network consistently demonstrates optimal performance, with accuracy rates of 99.5%, 99.2%, and 98.9% for Datasets A, B, and C, respectively. The ability of this type of classifier to learn complex relationships and patterns within large datasets, thanks to its large number of adjustable parameters, may be a factor in these results, as it is capable of detecting patterns and textures that are difficult to perceive and that characterize steatosis.

The SVM and RF classifiers, which achieve 99.1%, 99.0%, and 98.6% and 99.0%, 95.6%, and 98.2% for Datasets A, B, and C, respectively, are particularly well-suited to high-dimensional contexts, such as medical images, which exhibit a lot of characteristics.

While they do not compete with the previously mentioned classifiers, the decision tree, gradient boosting, and KNN classifiers have consistently demonstrated robust results, with an average above 90%. Both GB and DT are capable of addressing non-linear relationships between medical data without the necessity for complex pre-processing. In contrast, the KNN classifier can make predictions based on the similarity of the data, which allows it to identify similar local patterns between the images. This proved to be an effective approach for differentiating between the two classes.

A comparison of the datasets reveals that, in general, Dataset A, comprising ultrasound images, produces the most favorable results, even though this difference is not relevant in some cases. In contrast, Datasets B and C, comprising elastography images and ultrasound images in conjunction with elastography, do not exhibit a significant divergence in performance. The discrepancy between Dataset A and B, in some cases, may be attributed to the latter’s reduced dataset in comparison, which is insufficient to attain results as precise and reliable as the former, using specific classifiers such as RF and KNN. However, this is not the sole factor. A comparison of the results of Dataset C with those of Dataset A reveals that the former is unable to achieve the same level of performance as the latter. The combination of several types of images may be advantageous for better generalization and a lower probability of overfitting; however, given the results, using two types of images to train the models appears to not be as good as using just one, probably due to the increase in model complexity.

### 4.2. Deep Learning Approach

This section presents the results of tests carried out on five convolutional neural network architectures (DenseNet-201, Inception-ResNet-v2, ResNet50V2, VGG16, and VGG19), varying the optimizers and loss functions used, and understanding how attention mechanisms affect the models. In order to train and test the chosen architectures, different data augmentation techniques were applied to the images, in particular, rotations at different angles between 90° and −90°, horizontal and vertical inversions, as well as image enlargements. Similarly to the previous section, the procedure initially chosen was an exploratory analysis, carried out for testing purposes only. The loss function chosen was the cross-entropy loss as well as Adam as the optimizer. The number of training epochs was explored first, being initialized at 50. All convolutional neural network architectures were compared using transfer learning, freezing their layers and adding new layers to be trained for the problem in question.

#### 4.2.1. Number of Epochs

All the models were trained for 50 epochs in order to determine the ideal number for training each of them. Table 3 shows the best result obtained by each of the models throughout the training process and in which epoch that result was obtained.

Looking at Table 3, it can be seen that all architectures, always reach convergence after 30 epochs, although it is more frequent to happen closer to the 40 epochs. The fact that the best results are obtained towards the end of training may indicate that the data have very complex patterns that are difficult to detect and take time to characterize properly. This complexity also seems to be reflected in the results obtained: for Dataset B, the F1 score is no more than 73%, while for Dataset C, the maximum is 76%. For Dataset A, the VGG16, ResNet50V2, and DenseNet-201 architectures already exceed the 80% threshold, with the latter achieving an F1 score of 87.5%, 2.5% more than the second-best architecture. DenseNet-201 can propagate complex features due to its connectivity pattern between all layers, which may be at the heart of this superior performance in the case of Datasets A and C. For Dataset B, ResNet50V2 achieves the best performance with 72.7%.

Comparing the overall results of each dataset, it can be seen that both Datasets B and C do not achieve the same results as Dataset A. Perhaps the elastography patterns are more difficult to characterize using these architectures and parameters combination and make it more difficult for the model to generalize. In other words, at this stage, the ultrasound dataset is capable of producing the best results, even if they do not reach the 90% level where a neural network can be considered quite reliable.

#### 4.2.2. Optimizers

**Figure 11:**
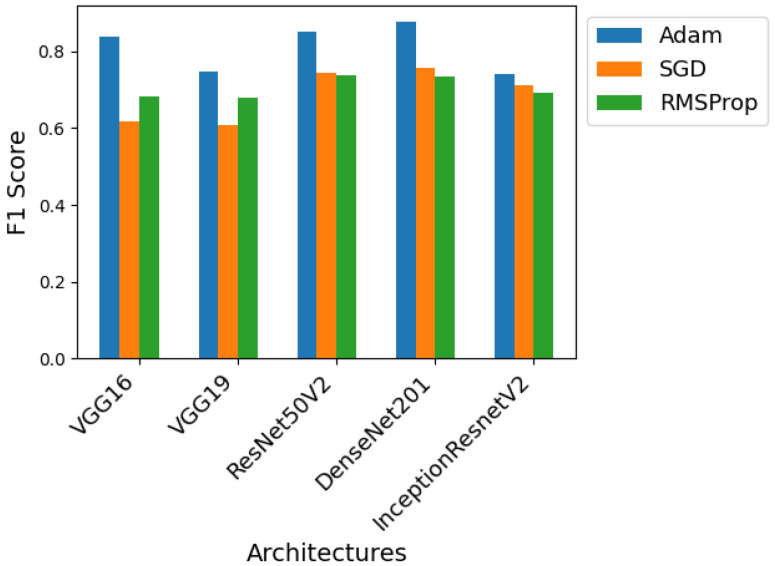
Performance of the various neural network architectures, using three different optimizers, for Dataset A.

**Figure 12:**
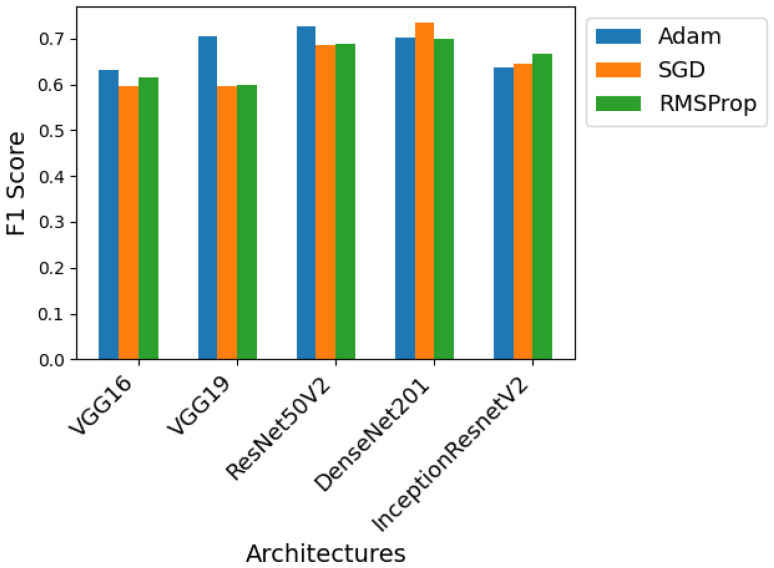
Performance of the various neural network architectures, using three different optimizers, for Dataset B.

**Figure 13:**
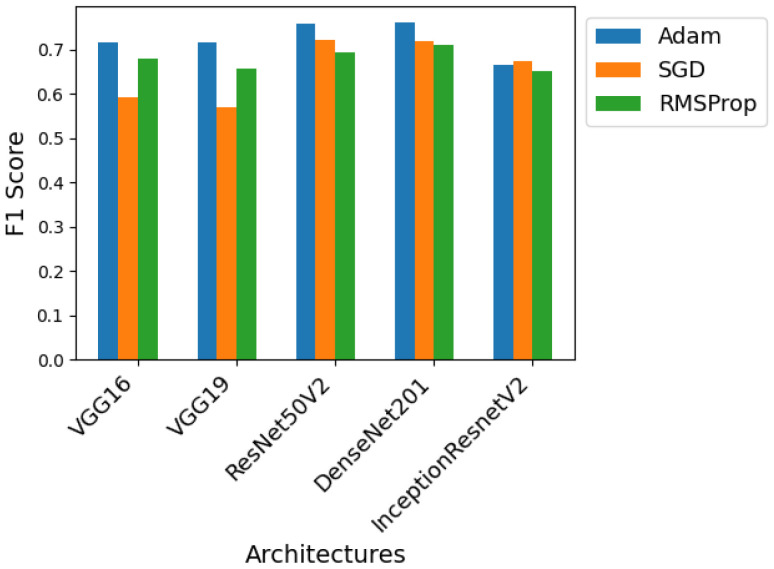
Performance of the various neural network architectures, using three different optimizers, for Dataset C.

Using the ideal number of epochs for each architecture found in the previous test and based on the initial procedure, three different optimizers were tested. For each network architecture, three models were trained, one with the Adam optimizer, one with the gradient descent optimizer, and the last with the RMSprop optimizer. The best F1 score obtained for each of these models and optimizers is shown in the graphs from Figure 11, Figure 12 and Figure 13 so that we can compare the effect of the optimizers on each of the architectures.

For Dataset A, as can be seen in Figure 11, all architectures benefit from using the Adam optimizer, which always results in superior performance. Except for the Inception-ResNet-v2 architecture, where this optimizer shows a large performance gap compared to its competitors because it typically converges faster than them and is less sensitive to hyperparameter configurations.

Looking at Figure 12, which shows the performance of the models using Dataset B, the trend of the Adam optimizer being superior is not maintained for all architectures. In this case, DenseNet-201 and Inception-ResNet-v2 achieve the best performance using Stochastic gradient descent and RMSprop, respectively. The model combining SGD and DenseNet-201 is the one that achieves the best overall results. With this set of data and this architecture, the use of SGD proves beneficial, perhaps because it manages to overcome a local minimum where Adam may have become stuck.

Finally, looking at Figure 13, where Dataset C is used for training, the Adam optimizer remains superior in most cases. The only exception is the Inception-ResNet-v2 architecture, which this time benefits from the use of SGD. Again, in certain cases, the usage of the SGD optimizer can be advantageous, and this Dataset And architecture proves to be one of those cases.

In general, the ResNet50V2 and DenseNet-201 architectures produce the best results regardless of the optimizer used. The Inception-ResNet-v2 architecture is the only one to use a different optimizer for each dataset, Adam for Dataset A, RMSprop for Dataset B, and SGD for Dataset C. As it is the result of combining two different architectures, Inception and ResNet, it may be more adaptable to different optimizers, justifying this deviation from the norm.

#### 4.2.3. Loss Functions

**Figure 14:**
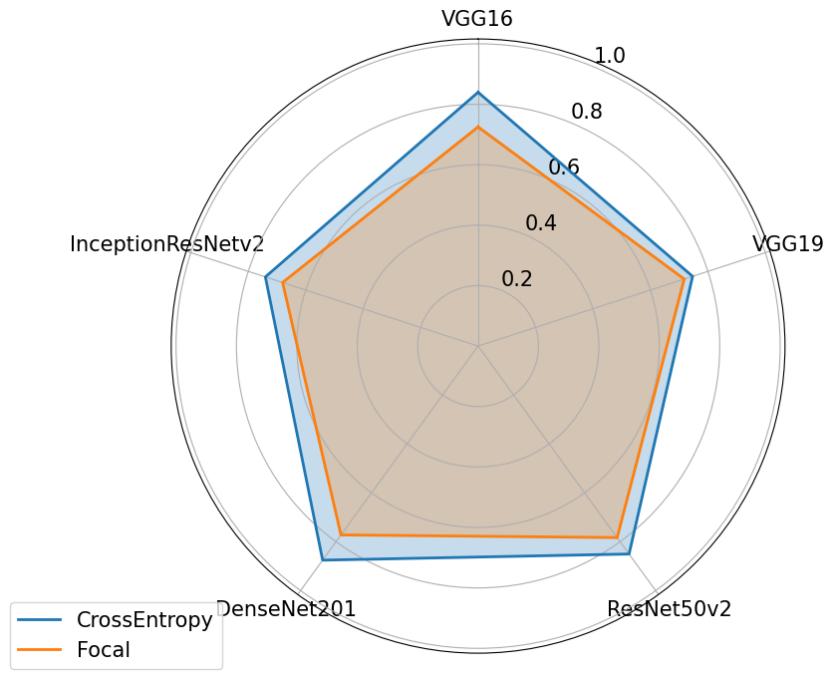
Performance of the various neural network architectures, using two different loss functions, for Dataset A.

**Figure 15:**
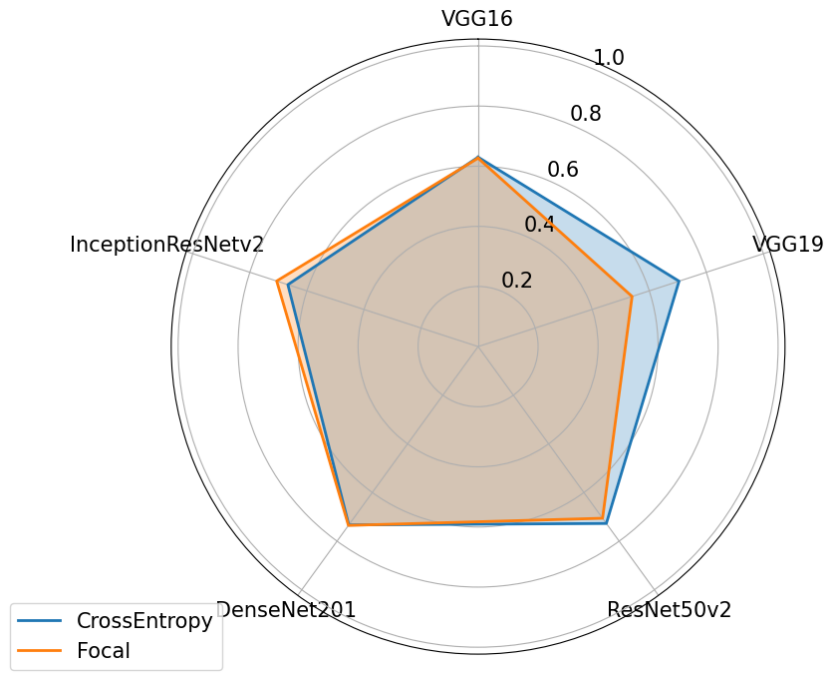
Performance of the various neural network architectures, using two different loss functions, for Dataset B.

**Figure 16:**
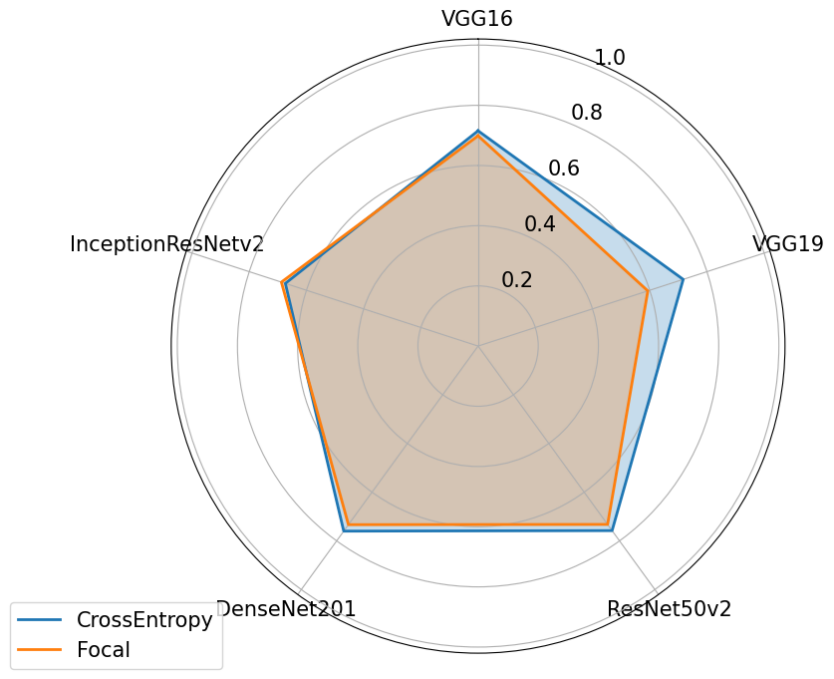
Performances of various neural network architectures, using two different loss functions for Dataset C.

After the optimizers were tested, two different loss functions were compared, namely, the cross-entropy loss and the focal loss. Using the parameters tested and determined in the previous sections, the results shown in Figure 14, Figure 15 and Figure 16 were obtained.

The cross-entropy loss function, as shown in Figure 14, gives better results in all cases. Focal loss is more advantageous in the case of unbalanced data, as it gives more weight to examples that are more complex to classify. Since measures have been taken to deal with this problem, the use of this function proves to be of little help in this case.

For Dataset B (Figure 15), the cross-entropy loss appears to be better for only 3 of the architectures, with VGG19 showing the largest difference and the difference being almost negligible for ResNet50VS and VGG16. For the other two architectures, the focal entropy loss appears to be better, with the difference being almost insignificant for the DenseNet-201 architecture. Inception ResNet, contrary to what was observed in Figure 14, improved with the use of the focal loss function this time. Although focal loss is more advantageous in the case of unbalanced data, it can contribute to more efficient model training, resulting in a better generalization capacity even in balanced sets.

Again, for Dataset C, as seen in Figure 16, the cross-entropy loss function gave the best performance in all but one case. This time, for the Inception architecture, the focal loss was superior to the cross-entropy. As this is the only one that uses the SGD optimizer, unlike all the others that use Adam, this may be the reason for this divergence from the trend. Dataset B also showed this deviation and again the optimizer used by this architecture is not Adam. Different optimizers can work better with different loss functions, in this case, Adam always seems to work better with cross-entropy and the other optimizers work better with focal loss. However, as the difference between the two functions is noticeable in most cases, cross-entropy is clearly the better option. This function has the advantage of penalizing misclassifications made with high confidence and also rewarding correct classifications with high confidence, providing a consistent approach that works well in most cases.

#### 4.2.4. Attention Mechanism

**Figure 17:**
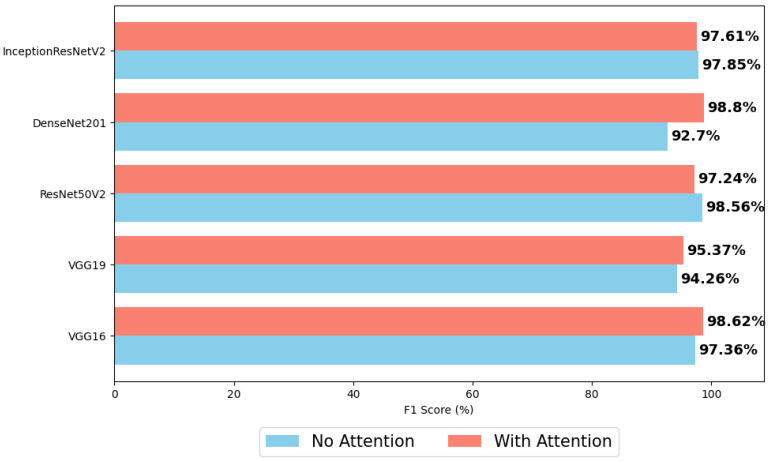
Performances of the various neural network architectures, with and without attention, for Dataset A.

After three different tests, the results were not satisfactory and relevant, so an attempt was made to determine what was hindering the performance of the models. After several attempts to solve the problem, it was concluded that the problem lay in the way the data augmentation was being performed, so the ImageDataGenerator [39] algorithm was replaced by an Albumentations augmentation pipeline [40]. Further investigation revealed that the default fill mode of the TensorFlow function is different from that of the Albumentations functions. The latter uses interpolation so that when the image is rotated, the “new” corners are filled with a version of the image before the change, creating image continuity. Whereas ImageDataGenerator() just creates a sort of blur effect. A fill mode such as that of the ImageDataGenerator is not ideal, because if the rotation is greater it can introduce a lot of noise, which can hinder the model’s learning of features and patterns. Sometimes the introduction of noise can be used in an attempt to create a more robust model, in which case it could compromise the results. On the other hand, how the Albumentations library is populated does not seem to cause any friction in the learning of the models, as it does not alter the integrity of the images. With this change, the results changed considerably and the new F1 score values for each architecture, for Dataset A, are shown in Figure 17 as the values obtained without using attention. Assuming that the change in the data augmentation technique is only to improve the results, it can also be expected that the conclusions drawn from the previous results are maintained with this change.

This section compares architectures with and without a multi-head attention mechanism.

Figure 17 shows that the addition of the attention mechanism for ultrasound images improves the performance of four of the six models. The VGG16 and VGG19 architectures show a negligible performance improvement of close to 1%, achieving F1 scores of 98.62% and 95.37%, respectively. The DenseNet-201 architecture, on the other hand, improves significantly with this addition, proving that attention has managed to synthesize and collect relevant information in the different heads, improving the model’s convergence and generalization capabilities. DenseNet-201 achieves the highest F1 result of this dataset (98.8%). On the other hand, ResNet50V2 with an attention mechanism performs worse, with results ranging from 98.56% to only 97.24%. The Inception-ResNet-v2 architecture also performs worse when multi-head attention is used, but the difference is practically negligible.

Moving on to analyze Dataset B, the Inception-ResNet-v2 and ResNet50V2 architectures still do not benefit from the use of an attention layer, with the latter standing out, where the performance without attention exceeds the performance with attention by 6.23%. Sometimes the introduction of attention mechanisms can increase the complexity of the network, making it more difficult to generalize. Furthermore, the addition of attention can sometimes introduce distractions or noise that impair the model’s ability to grasp patterns. In the case of the DenseNet-201, VGG16, and VGG19 architectures, attention helps to understand complex patterns and generalize the model, all of which improve with use. DenseNet-201 again shows a very large improvement, from 94.83% to 97.98%, which may indicate synergy between this architecture and multi-head attention in this particular classification task.

The results for Dataset C confirm the trends seen in Dataset A and B. Both VGG architectures improve, although not significantly, as does ResNet-101. Once again, DenseNet-201 is the one that improves the most, achieving an F1 score of 83.2%, but performing well below the others. Perhaps because this dataset has two types of data from two different imaging methods, DenseNet is not as good at understanding two additional different patterns. The Inception architecture never seems to work well with the use of attention, as its performance always deteriorates, although this deterioration is never very significant. On the other hand, with ResNet50V2, multi-head attention always leads to a much worse result. There may be some interference from this type of mechanism with this particular architecture.

In conclusion, each architecture responds differently to the integration of attention mechanisms, specifically multi-head attention; therefore, those that benefit from its use will continue to incorporate it, while those that do not will proceed without it.

#### 4.2.5. Final Evaluation

The best performance of each architecture for the three datasets is shown in Table 4. In this evaluation, the configuration chosen in the previous comparisons has been used to obtain the best possible performance. The overall performance of each dataset was also evaluated in an attempt to understand which is the most advantageous.

The highest score was achieved by the model using Dataset A and the DenseNet-201 architecture with a score of 98.80%. For Dataset B, the same architecture produces the second-best result within this dataset, reaching 97.98%. However, Dataset C’s performance is much lower than the others, with a score of 83.20%. DenseNet-201 is an architecture characterized by its densely connected structure, where each layer receives inputs from all previous layers. It manages to capture complex relationships between data and can achieve excellent results, as in the case of the ultrasound and elastography sets. However, this complexity can hinder its performance on the mixed dataset. As the patterns found in the ultrasound images may differ from those found in the elastography images, they may contribute to a possible model instability in some situations. The model will probably need further parameter optimization to better understand these patterns and improve generalization.

For Datasets B and C, the ResNet50V2 architecture achieves the highest metrics, with F1 values of 98.23% and 97.58%, respectively. Dataset A reaches almost the same level as the aforementioned architecture with 98.56%. One of the main features of this network may be at the heart of this optimal performance: its identity connections. These connections make it possible to preserve information from earlier layers and help overcome the problem of gradient dissipation.

The VGG16 and VGG19 architectures also perform very well in this context. On Dataset B, their performances are similar at 95.86% and 95.40%, respectively. However, for the other two sets, VGG16 is superior, achieving metrics of 98.62% and 96.96% for Datasets A and C, compared to 95.37% and 90.66% for VGG19. This difference illustrates the better generalization capacity of the first architecture, which, because it is simpler (it has three fewer convolutional layers), ends up being easier to tune and more robust for smaller datasets.

Finally, the Inception-ResNet-v2 architecture performs quite well for all datasets, never offering the best performance but not deviating too far from the norm. The F1 scores obtained for Datasets A, B, and C are 97.85%, 96.46%, and 94.53%, respectively. As mentioned above, the result is a combination of two different architectures, which makes it very versatile and consistent.

Analyzing the different datasets And comparing them, the set that gives the best results is Dataset A, followed by Dataset B and then Dataset C, with the highest performance percentages obtained in these sets being 98.80%, 98.23%, and 97.58%, respectively. Although the results obtained with ultrasound data are almost always higher than those obtained with elastography, the difference is never too significant. Taking into account the best results obtained with each dataset, as mentioned above, this insignificant difference is noticeable, indicating that elastography is ultimately on par with ultrasound in terms of diagnostic capacity.

The use of a mixed dataset is never advantageous, although it can make the models more robust, because it never gives the best result and, on some architectures, it significantly worsens performance.

## 5. Conclusions

This study presents a comparative analysis using traditional machine learning and deep learning models applied to the detection and classification of liver steatosis to evaluate their usefulness in the diagnosis of this disease, as well as to understand the relevance of elastography and ultrasound techniques in this context.

To this end, a series of machine learning classifiers and deep learning architectures were selected and trained using datasets of ultrasound and elastography images, as well as a dataset that was a mixture of both. In order to achieve the best possible performance of these models, several techniques were tested, including sliding window parameter testing, variations in the number of features, optimizers, loss functions, and the use of attention mechanisms.

The machine learning models showed promising results, with all six classifiers consistently achieving F1 scores above 90% across all datasets used. These results prove the value of machine learning algorithms employed in liver steatosis diagnosis. Moreover, the neural network classifier, which achieves the best results in this study, demonstrates the high performance that algorithms can reach when using all three sets of data. Specifically, it achieves F1 scores of 99.5%, 99.2%, and 98.9% for the ultrasound, elastography, and mixed image datasets, respectively.

In terms of the deep learning architectures tested, all five achieved results higher than 90%. Out of all the models, DenseNet-201 was the one that scored the highest mark in general, with a 98.80% F1 score using ultrasound images. However, coming really close, with an F1 score of 98.56% in the latter dataset, the ResNet50V2 was the architecture that performed the best overall taking all the datasets into account. For Dataset B and C, the mentioned model obtains scores of 98.23% and 97.58% in this order. Once more, the stated results imply a highly prepared dataset of algorithms to assist in the medical assessment of steatosis and possibly other associated liver diseases.

When comparing the two approaches based on the results from the datasets used, it is clear that the machine learning methods outperformed the deep learning methods. In addition to the neural network model, both the random forest (99.0% for Dataset A and 98.2% for Dataset C) and the support vector machine (99.1% for Dataset A, 99.0% for Dataset B and 98.6% for Dataset C) surpass the performances of all deep neural networks in almost all cases. Although machine learning algorithms can perform better with a lower number of data and are less complex, improving interpretability and speed of implementation (very important in the medical context), since the best performances of the two methods are really close, deep learning cannot be discarded as a useful technique in the diagnosis of steatosis, as it can extract its own features and generate models that are more robust to changes in the patterns and relationships in the data. It is also worth noting the emerging trend of obtaining and storing more data at a time, which can be a point in favor of deep learning algorithms.

The results from the ultrasound image dataset confirm that ultrasound is the preferred imaging method for diagnosing steatosis, though the advantage is not substantial. In the majority of cases for deep learning, and some cases for machine learning, elastography comes very close to the ultrasound results, proving that the former can be practically as reliable as the latter in assessing fatty liver disease. Furthermore, although the dataset of images containing data from both imaging techniques can perform quite well, it consistently ranks as the worst-performing of the three. This may be due to the added complexity of categorizing patterns and relationships across two different modalities. With this in mind, and considering the potential for more robust models, the use of only one imaging modality is suggested.

This research provides valuable insights into the field of medical image classification and advocates for the integration of advanced machine learning and deep learning technologies in medicine, specifically in diagnosing liver diseases. The promising results open the way for future studies aimed at optimizing these models and exploring their applications in other medical domains.

## Figures and Tables

**Table 1:** Hyperparameters of the selected classifiers.

Classifier	Parameter	Value
Decision tree	criterion	‘gini’
	max_depth	14
Gradient boosting	n_estimators	121
	learning_rate	1.32
KNN	n_neighbors	17
	leaf_size	10
	p	5
Neural network	hidden_layer_sizes	100
	activation	‘tanh’
	learning_rate	‘invscaling’
	max_iter	861
RF	n_estimators	10
	criterion	‘gini’
SVM	C	6000
	Kernel	‘rbf’

**Table 2:** Performance (F1 score) in the percentage of each classifier with its best configuration for each of the datasets (A, B, and C).

	DT	GB	KNN	NN	RF	SVM
**Dataset A**	91.8	95.8	96.0	99.5	99.0	99.1
**Dataset B**	88.9	93.8	90.8	99.2	95.6	99.0
**Dataset C**	90.5	93.7	92.2	98.9	98.2	98.6

**Table 3:** Best F1 score value obtained during training and the epoch at which this value was obtained for each type of architecture and each dataset.

Architecture	Dataset A		Dataset B		Dataset C	
	**F1 Score (%)**	**Epoch**	**F1 Score (%)**	**Epoch**	**F1 Score (%)**	**Epoch**
VGG16	83.9	40	63.0	47	71.5	33
VGG19	74.6	39	70.4	38	71.7	39
ResNet50V2	85.0	32	72.7	41	75.8	42
DenseNet-201	87.5	32	70.2	34	76.0	46
Inception-ResNet	74.0	48	63.8	47	66.7	49

**Table 4:** Performance (F1 score) percentage of each architecture with the best configuration for each dataset (A, B, and C).

Architecture	Dataset A	Dataset B	Dataset C
VGG16	98.62	95.86	96.96
VGG19	95.37	95.40	90.66
ResNet50V2	98.56	98.23	97.58
DenseNet-201	98.80	97.98	83.20
Inception-ResNet-v2	97.85	96.46	94.53

## Data Availability

The public database “Dataset of B-mode fatty liver ultrasound images” [16] was used, which includes images from 380 patients with steatosis and 170 healthy individuals. The second set of images was acquired for this study and consists of 96 elastography images, 61 of which are from NAFLD patients and the remaining 35 from healthy subjects.
